# Treatment of Pyorrhea Alveolaris

**Published:** 1907-02

**Authors:** Gustavus North

**Affiliations:** Cedar Rapids, Iowa.


### TREATMENT OF PYORRHEA ALVEOLARIS

By Gustavus North, A. M., D. D. S., Cedar Rapids, Iowa.

Pyorrhea alveolaris is one of the hardest diseases to control that comes before the dental practitioner. Some consider it a local disease, dependent upon some micro-organism; others consider it as arising from constitutional disturbances. It certainly depends upon both conditions as causitive factors. Some constitutional disturbances prepare the way for micro-organism. Local deposits assist in destroying the surrounding tissue; but this of itself could not produce pyorrhea, as in some cases we have very little foreign substance upon the roots of the teeth. Sometimes the gum appears almost in a normal condition, but far up under the gum we find the alveolar process gradually wasting away, caused by some destructible element. Again we find little granulations of lime salt upon the roots of teeth irritating the gum and surrounding tissue, causing swelling and sloughing of the parts. This local disturbance alone is not the cause of pyeorrhœa.

Deposits of tartar around the teeth is not pyorrhœa alveolaris; it can be removed and treated locally.

Without systematic treatment true pyorrhœa cannot be cured, especially if a gouty diathesis due to abundance of uric acid or low vitality is present. In my observations I find such symptoms existing.

After all deposits have been removed and the gums properly treated, I administer "Sal-Hepatica" from one to two teaspoonfuls (according to condition of patient) dissolved in a tumblerful of water each morning about thirty minutes before breakfast. It is effervescent, saline laxative and uric acid solvent.

I have had wonderful success with the above treatment.

